# GA-Hecate antiviral properties on HCV whole cycle represent a new antiviral class and open the door for the development of broad spectrum antivirals

**DOI:** 10.1038/s41598-018-32176-w

**Published:** 2018-09-25

**Authors:** Mariana Nogueira Batista, Paulo Ricardo da Silva Sanches, Bruno Moreira Carneiro, Ana Cláudia Silva Braga, Guilherme Rodrigues Fernandes Campos, Eduardo Maffud Cilli, Paula Rahal

**Affiliations:** 1 Institute of Bioscience, Language and Exact Science, UNESP - São Paulo State University, São José do Rio Preto, SP Brazil; 2 Institute of Chemistry, UNESP – São Paulo State University, Araraquara, SP Brazil

## Abstract

In recent years, synthetic peptides have been considered promising targets for drug development that possess low side-effects, are cost-effective and are susceptible to rational design. Hecate was initially described as a potent bacterial inhibitor and subsequently as an anticancer drug with functions related to its lipid interaction property. Viruses, such as hepatitis C virus (HCV), have a lipid-dependent life cycle and could be affected by Hecate in many ways. Here, we assessed modifications on Hecate’s N-terminus region and its effects on HCV and hepatotoxicity. Gallic acid-conjugated Hecate was the most efficient Hecate-derivative, presenting high potential as an antiviral and inhibiting between 50 to 99% of all major steps within the HCV infectious cycle. However, the most promising aspect was GA-Hecate’s mechanism of action, which was associated with a balanced lipid interaction with the viral envelope and lipid droplets, as well as dsRNA intercalation, allowing for the possibility to affect other ssRNA viruses and those with a lipid-dependent cycle.

## Introduction

Peptides have represented a new therapeutic approach for many human diseases, such as cancer, metabolic diseases, Alzheimer’s disease and infectious diseases, including viral diseases^1,2^. Peptide drugs are an advantageous source for new antiviral treatments, which possess low side-effects, rapid elimination following treatment, can be produced in a cost-effective way and are susceptible to rational design, representing a virtually inexhaustible source of directed-modifications^3–5^. In addition, cationic peptides are commonly produced as part of the innate immune response against infections^6^, representing an evolutionary strategy and therefore an effective way to keep infections under control.

Hecate is a well-known synthetic cationic peptide that forms amphipathic α-helices and was previously described as anti-bacterial and has subsequently been used for anticancer treatment^7–9^ showing a well-described interaction with membranes. The suggested mechanism by which Hecate interacts with membranes is through intercalation in the phospholipid/glycerol backbone, followed by chemical instability and membrane disruption^10^. These properties could be used to affect viruses with some lipid dependence, which is a characteristic of a wide range of viruses. A viral cycle with well-known lipid dependence is the hepatitis C virus (HCV) infectious cycle. HCV is an enveloped virus, and its life cycle is intimately associated with lipid metabolism^11^. HCV is a lipo-viral particle, which uses lipid-related factors for entry, including glycosaminoglycans (GAGs), low-density lipoprotein receptor (LDL-R), scavenger receptor class B type I (SR-BI) and likely Niemann-Pick C1-like 1 cholesterol absorption receptor (NPC1L1)^12^. HCV replication/assembly is associated with cytosolic lipid droplets (LD), and its release overlaps the very low-density lipoprotein pathway^11–14^.

Hepatitis C is an infection that is frequently chronic and progressive and is caused by hepatitis C virus^15^, and it is responsible for approximately 399,000 deaths per year. HCV infects about 1% of the world population and costs millions of dollars yearly for the treatment of chronic patients. There is no a preventive vaccine against HCV, and the current treatment, including the second and third generation of direct-acting antivirals (DAAs) for non-structural proteins, are high cost and not accessible for most patients. In addition, this treatment keeps generating drug-resistant virus, as well as presenting side-effects, although at lower levels than in previous treatments. Therefore, regarding economics and potency, new and more efficient antiviral options are still necessary. The aim of this study was to clarify the hypothesis of Hecate as an antiviral, testing the effects of Hecate and its derivatives on the HCV infectious cycle and its peptide modifications on hepatocyte viability.

## Results

### Viability and antiviral effect of Hecate and its derivatives on HCV subgenomic replicons

**Figure 1:**
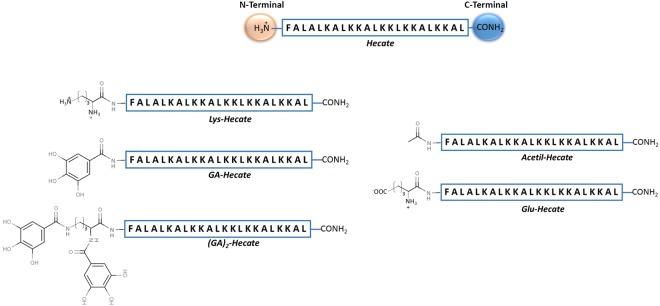
Schematic representation of the N-terminus modifications of Hecate.

**Figure 2:**
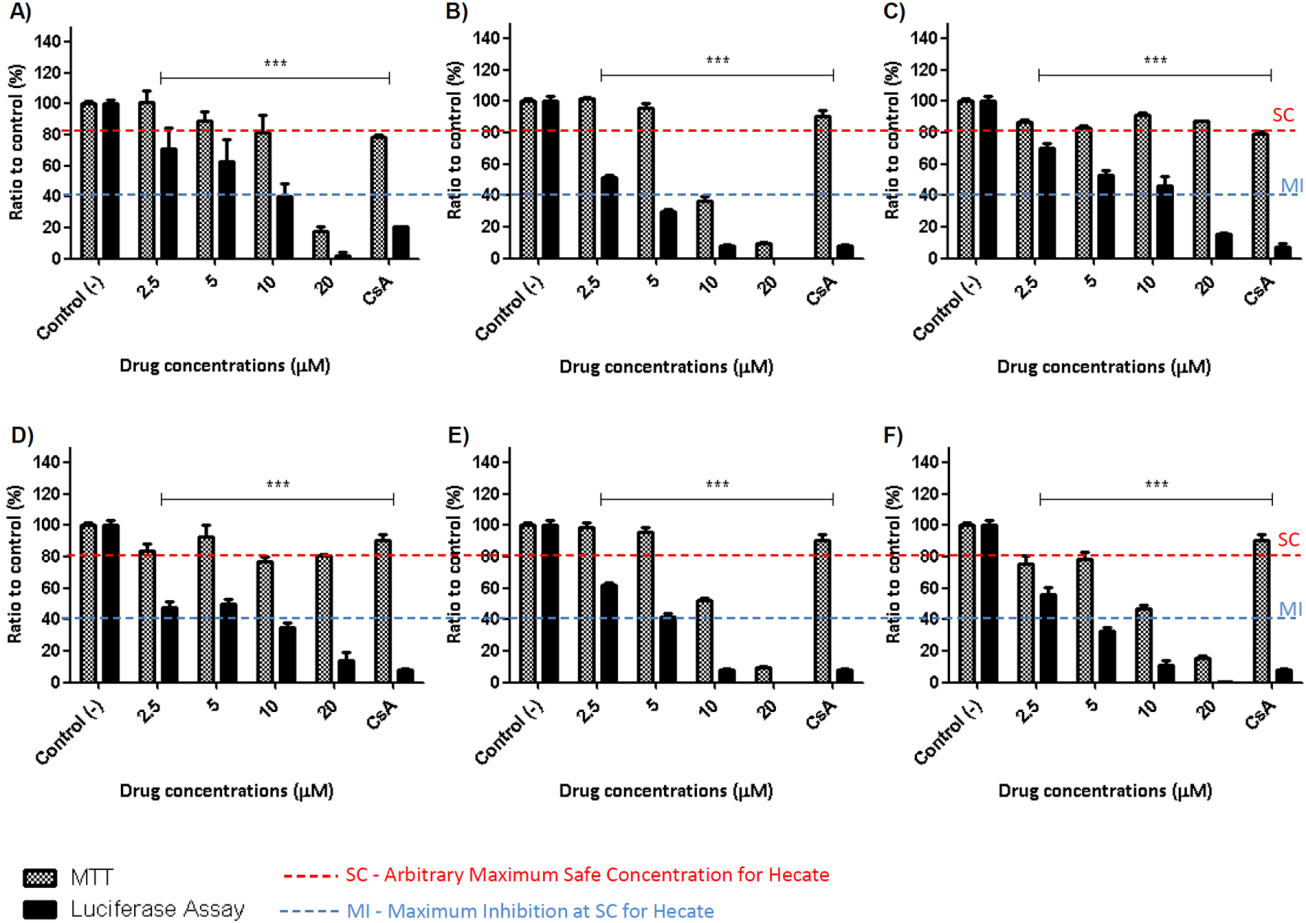
Hecate and its derivative-peptide effects on subgenomic HCV genotype 2a replication and hepatotoxicity. (**A**) Hecate. (**B**) Lys- Hecate. (**C**) GA-Hecate. (**D**) GA_2_-Hecate. (**E**) Acetyl-Hecate. (**F**) Glu-Hecate. Luciferase assays report HCV replication and MTT assays report the viability of Huh-7.5 cells harboring SGR-JFH-1 Feo under peptide treatment. Water was used as a negative control of virus replication inhibition and cell death (Control (−)), and Cyclosporine A was used as a positive control for virus replication inhibition (Control (+)). Red dotted lines represent the arbitrary threshold of 80% cell viability, and blue dotted lines represent the maximum inhibition presented by Hecate at the maximum safe concentration. Bars represent triplicates of the three independent assays. ***P < 0.01 vs control (−).

To understand the effect of Hecate on HCV and host cells, we tested the original structure of Hecate and the structures with modifications at the N-terminus. A schematic representation of the modified peptides is presented in Fig. 1, and the physicochemical properties along with the biological activities are summarized in Table 1. Considering the arbitrary threshold of 80% cell viability, Hecate reached its maximum non-toxic concentration in hepatocytes at 10 μM and inhibited approximately 60% of HCV replication (Fig. 2A). The addition of a positive amino acid residue (Lys) to Hecate (Lys-Hecate), reduced the safest concentration to 5 μM, inhibiting approximately 75% of the HCV replication (Fig. 2B). GA-Hecate and GA_2_-Hecate reduced cell toxicity, and at 20 μM showed 70% (Fig. 2C) and 80% of cell viability (Fig. 2D), respectively. GA-Hecate and GA_2_-Hecate also had reduced inhibition efficiency in HCV replication when compared with Hecate, showing a half maximum effective concentration (EC50) of 11.18 μM and 34.34 μM, respectively, while Hecate was 8.57 μM. However, as these peptides could be used in higher, safe concentrations, they inhibited approximately 80% of virus replication inhibition at the maximum safe concentration. Peptides Glu-Hecate and Acetyl-Hecate presented higher toxicity than Hecate, with both reaching a maximum safe concentration at 5 μM and inhibiting approximately 65% of virus replication at this concentration (Fig. 2E,F). Considering that GA-Hecate presented the best SI (3.13), it was used for further analysis. To better understand, GA-Hecate ability to inhibit HCV, it was evaluated at 24, 48 and 72 h post-treatment (see Supplementary Fig. S1). The treatment showed a time and dose-dependent effect. The EC50 and CC50 72 h post-infection were 3.8 µM and 19 µM respectively and therefore, the SI was increased to 5. Considering that there is a balance between toxicity and virus inhibition, we further investigated the cause of cell death. In concentrations ≥20 μM there was some peptide-induced necrosis but it was not seen in concentrations lower than 20 μM (see Supplementary Figs S2, S3). There was a slight increase of apoptosis induction by GA-Hecate at 20 µM 72 h p.t but there was no significate apoptosis induction in any tested time point or concentration. Thus the proposed mechanism of action indicates a necessity of peptide aggregation for pore formation and cell death induction by necrosis, possible only at high peptide concentrations. On the other hand, at low concentrations, no cell death is triggered by GA-Hecate due to the absence of molecules enough to pores formation (see Supplementary Fig. S4).

**Table 1:** General characteristics and biological properties presented by Hecate and its N-terminus derivative cationic peptides.

Peptide	Sequence	Length	MW	Net charge	EC50^a^ (μM)	CC50^b^ (μM)	SI^c^
Hecate	FALALKALKKALKKLKKALKKAL	23	2535.6	+10	8.57	12.17	1.42
Lys-Hecate	K-FALALKALKKALKKLKKALKKAL	24	2664.5	+11	2.6	7.97	3.06
GA-Hecate	GA-FALALKALKKALKKLKKALKKAL	23	2688.4	+9	11.18	34.95	3.13
GA_2_-Hecate	GA2-FALALKALKKALKKLKKALKKAL	23	2969.7	+9	34.24	>100	2.92
Acetyl-Hecate	Acetyl-FALALKALKKALKKLKKALKKAL	23	2578.4	+9	3.79	9.43	2.53
Glu-Hecate	E-FALALKALKKALKKLKKALKKAL	24	2666.4	+9	3.30	9.12	2.76

^a^EC50 (50% effective concentration for HCV).

^b^CC50 (50% cytotoxic concentration).

^c^SI (selectivity index) was calculated using the ratio CC_50_/EC_50_.

**Figure 3:**
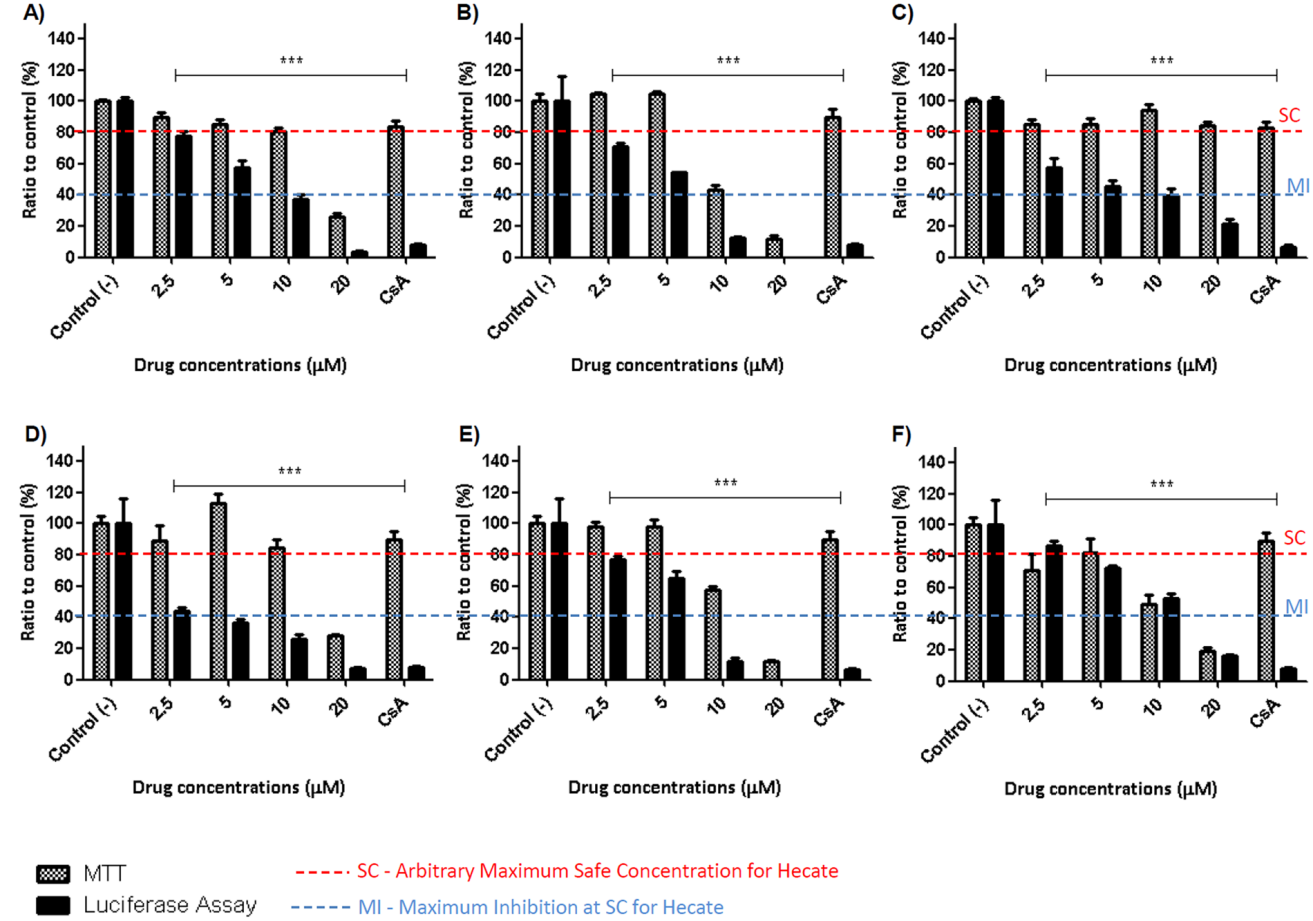
Hecate and its derivative-peptide effects on subgenomic HCV genotype 3a replication and hepatotoxicity. (**A**) Hecate. (**B**) Lys- Hecate. (**C**) GA-Hecate. (**D**) GA_2_-Hecate. (**E**) Acetyl-Hecate. (**F**) Glu-Hecate. Luciferase assays report HCV replication and MTT assays report the viability of Huh-7.5 cells harboring S52/SG-neo under peptide treatment. Water was used as a negative control of virus replication inhibition and cell death (Control (−)), and Cyclosporine A was used as a positive control for virus replication inhibition (Control (+)). Red dotted lines represent the arbitrary threshold of 80% cell viability, and blue dotted lines represent the maximum inhibition presented by Hecate at a maximum safe concentration. Bars represent triplicates of three independent assays. ***P < 0.01 vs control (−).

The same peptides and concentrations were tested in stable Huh-7.5 cell harbouring S52/SG-neo, a genotype 3 replicon. All peptides presented the same inhibition/toxicity profile for genotype 3; however, all peptides except GA-Hecate had reduced efficiency and lower tolerance in these cells than in that harbouring genotype 2a (Fig. 3). This peptide was also tested in a transient infection using the BM 4–5 Feo (genotype 1b) and also inhibited this genotype but in a lowest efficiency, reaching an inhibition of 60% in the maximum safe concentration (see Supplementary Fig. S5). Therefore, GA-Hecate was used to further analyse the HCV replication cycle.

### GA-Hecate effect on HCVcc

**Figure 4:**
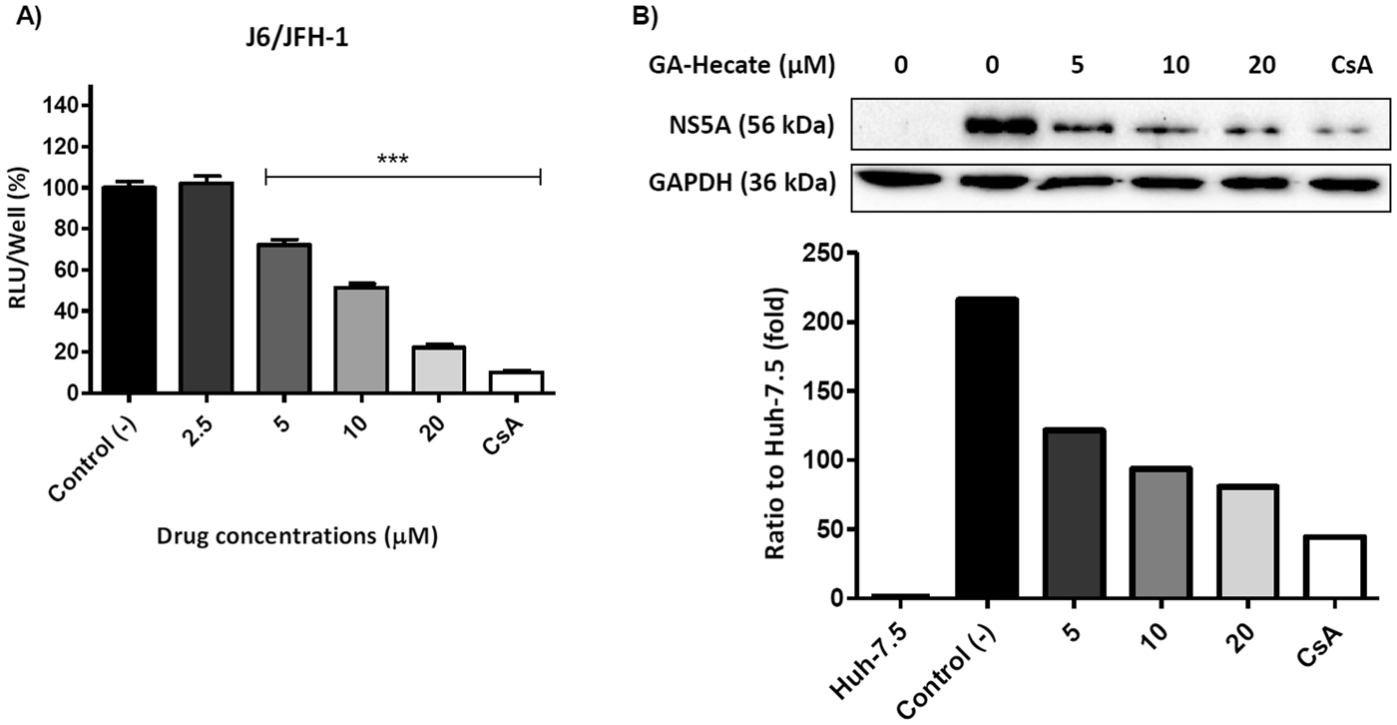
GA-Hecate effect on HCVcc replication. (**A**) HCV RNA expression measured by the Renilla luciferase reporter gene. (**B**) HCV non-structural (NS5A) protein expression. GAPDH was used as an endogenous control. Water was used as a negative control of virus replication inhibition (Control (−)), and cyclosporine A was used as a positive control of virus replication inhibition (Control (+)). Bars represent triplicate samples of three independent assays. RLU - Relative light units. ***P < 0.001 vs Control (−); F = 273.2, df (between columns) = 5.

**Figure 5:**
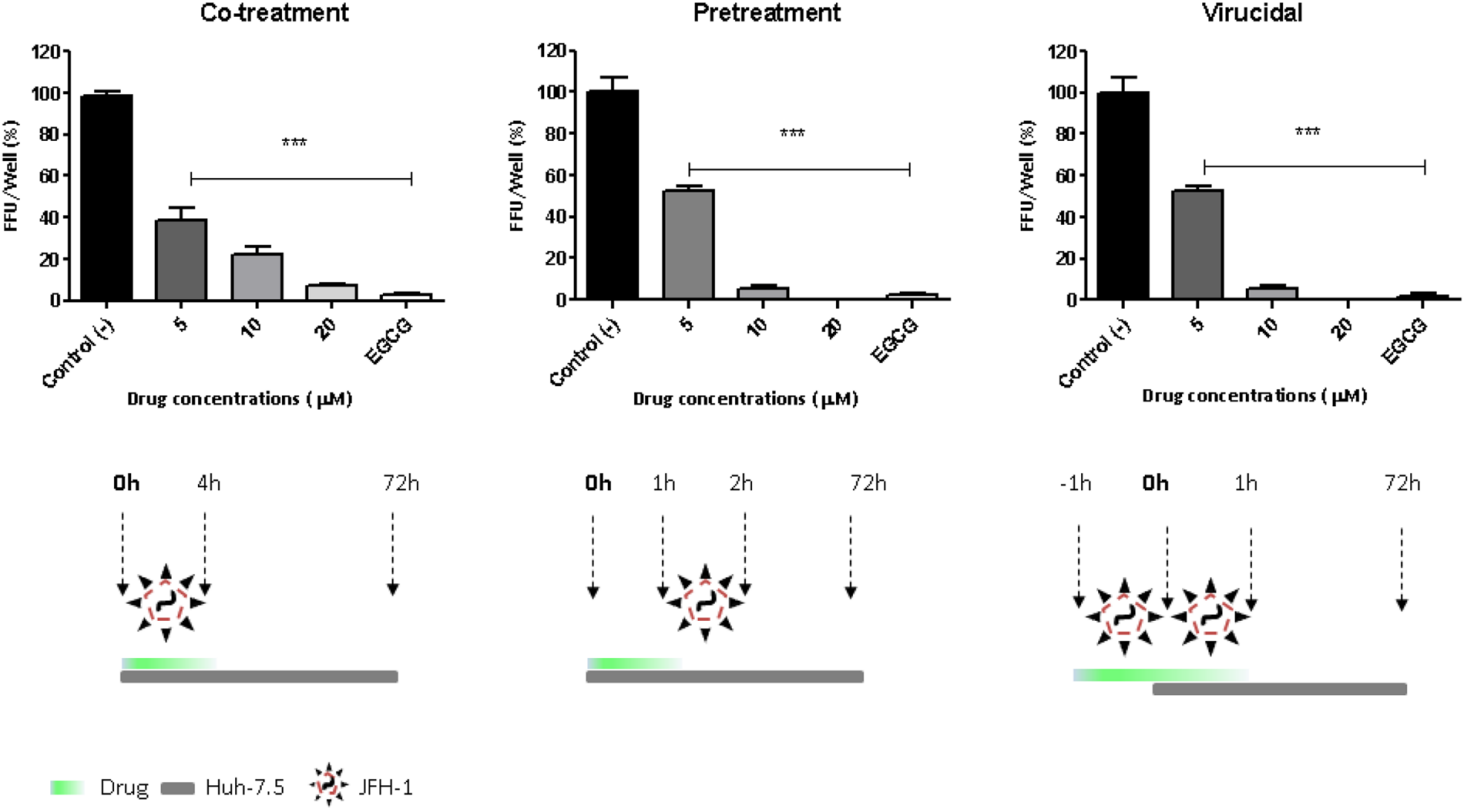
GA-Hecate effect on HCV entry. Co-treatment. Huh-7.5 cells were concomitantly infected and treated with GA-Hecate. The treatment was kept for 4 h and then removed. Treated cells were kept for an additional 72 h and submitted to IFI for NS5A. Bars represent triplicate samples of three independent assays. Epigallocatechin gallate (200 μM) was used as a positive control of HCV entry inhibition, and water was the negative control of HCV entry inhibition. ***P < 0.001 vs Control (−), F = 110.3, df (between columns) = 4. Pretreatment. The drug was added to the cells 1 h hour prior to the infection. Then HCVcc was added and maintained for an additional hour and at the end of 72 h, submitted to IFI. Bars represent triplicate samples of three independent assays. Epigallocatechin gallate (200 μM) was used as a positive control of HCV entry inhibition, and water was the negative control of HCV entry inhibition. ***P < 0.001 vs Control (−), F = 264.5, df (between columns) = 4. Virucidal. Drug and virus were incubated at 37 °C during 1 h and then added to the cells and kept for an additional hour. The inoculum was removed and cell were maintained during 72 h and submitted to IFI. Bars represent triplicate samples of three independent assays. Epigallocatechin gallate (200 μM) was used as a positive control of HCV entry inhibition, and water was the negative control of HCV entry inhibition. ***P < 0.001 vs Control (−), F = 264.5, df (between columns) = 4.

**Figure 6:**
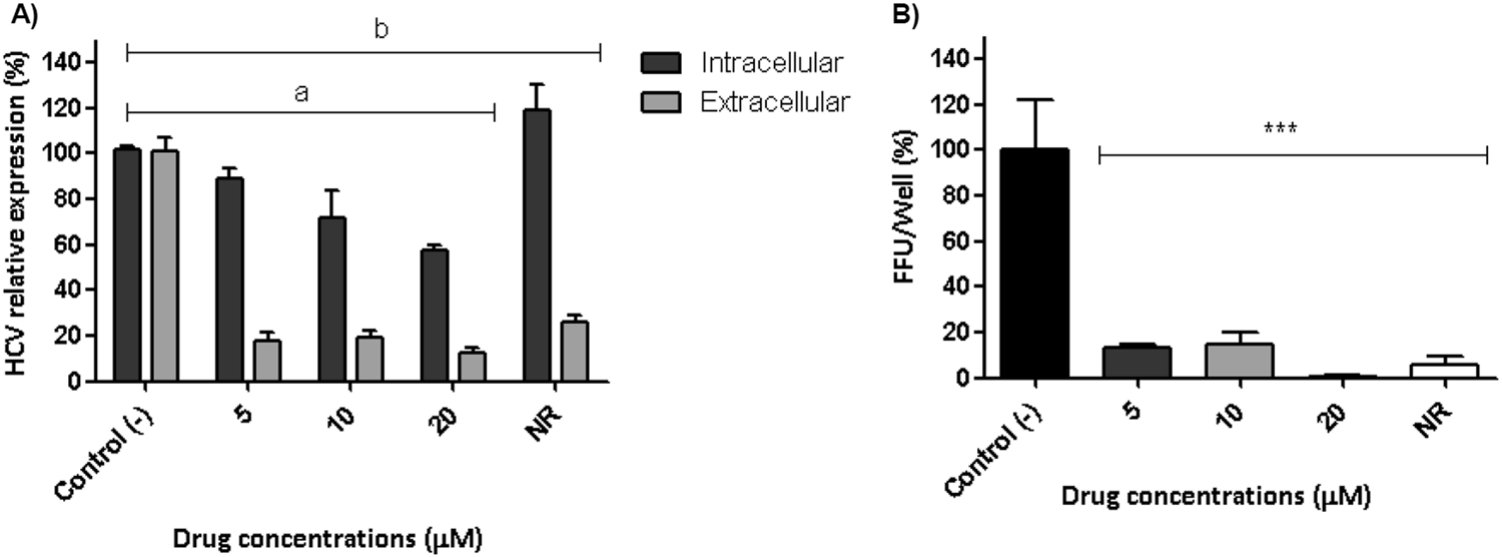
GA-Hecate effect on HCV Release and infectivity. (**A**) HCV-infected Huh-7.5 cells were treated for 24 h with GA-Hecate. RNA was isolated and summited to qPCR for HCV 5′ UTR. Intracellular HCV expression was normalized by GAPDH, and extracellular HCV expression was calculated by absolute quantification. The difference between the intra and extracellular RNA expression represents the HCV release. Bars represent triplicate samples of three independent assays. Naringenin (200 μM) was used as a positive control of HCV release inhibition and water was a negative control of HCV release inhibition. ^a^P < 0.001 vs Control (−), intracellular. ^b^P < 0.001 vs Control (−), extracellular. F = 22.58, df (row factor) = 4. (**B**) Infectivity. HCV-infected Huh-7.5 cells were treated for 24 h with GA-Hecate, and then the supernatant was collected and used to infect naïve Huh-7.5 cells. The capacity of HCVcc particles to generate a new infection after GA-Hecate treatment is represented by the bars. Bars represent triplicate samples of three independent assays. ***P < 0.001 vs Control (−). F = 16.4, df (between columns) = 4.

To evaluate GA-Hecate during the whole HCV infectious cycle, we used the HCV full-length replicons JFH-1 or FL-J6/JFH-50C19Rluc2AUbi. GA-Hecate inhibited approximately 85% of the HCV cell-culture derived viral particle (HCVcc) replication at 20 μM in a dose-dependent manner, considering the levels of viral RNA (Fig. 4A) and protein (Fig. 4B). GA-Hecate was also able to avoid virus entry in a dose-dependent manner; inhibiting 95% of virus entry at the maximum safe concentration and presenting a virucidal profile associated (Fig. 5). This peptide inhibited approximately 50% of the virus release, and this response was not dose-dependent, presenting this effect even at minimum concentration of 5 µM with no further improvement in increased concentration (Fig. 6A). To evaluate the viability of these released particles to generate a new infection, we evaluated the capacity of the infectious supernatant from the treated cells to generate infection in naïve Huh-7.5 cells since there is no more effective peptide in the supernatant (data not shown). The infectivity assay showed that the released particles were unable to generate a new infection, and presented approximately 100% infection inhibition at 20 μM (Fig. 6B).

### GA-Hecate mechanism of action in hepatocytes and mimetic membranes

**Figure 7:**
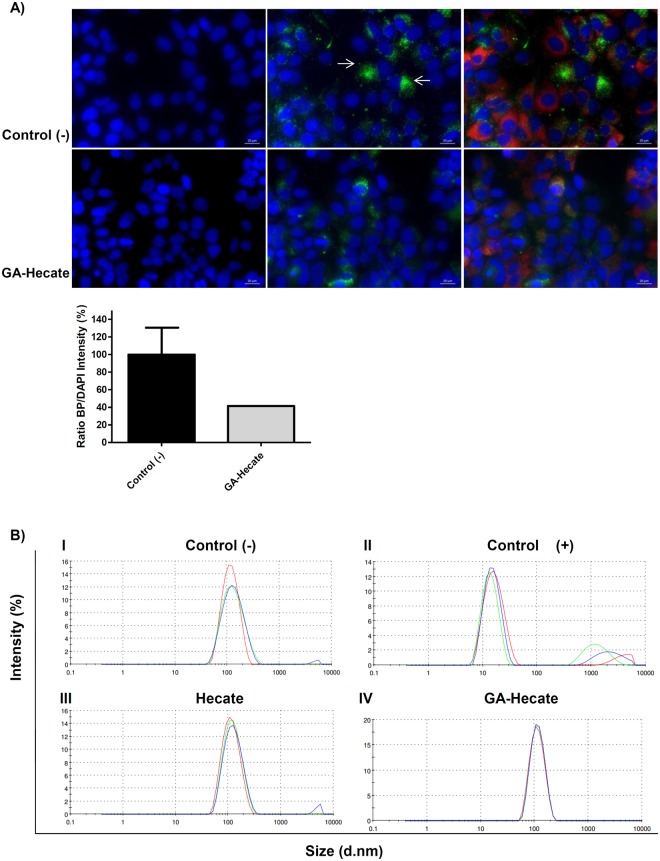
GA-Hecate effect on lipid droplets. (**A**) Pattern of lipid droplet distribution on treated and non-treated HCV-infected Huh-7.5 cells and the lipid droplets relative quantification. Blue: DAPI staining nuclei; Green: lipid droplets stained by Bodipy 493/503; Red: HCV NS5A represented by Alexa fluor-594. (**B**) Light Scattering Dynamic showing synthetic unilamellar vesicle size with lipid droplet constitution after treatment with the following: I. Water (negative control of size change); II. Triton-X 100 (positive control of size change); III. Hecate; IV. GA-Hecate.

To evaluate the capacity of GA-Hecate to interact with cytosolic lipid droplets, we first evaluated the droplets quantity and distribution in HCV-infected treated/non-treated cells. GA-Hecate altered the lipid droplets distribution and reduced its quantity (Fig. 7A). We further evaluated if Hecate could change the lipid droplets size by a dynamic light scattering assay; however, GA-Hecate does not alter lipid droplets size (Fig. 7B).

**Figure 8:**
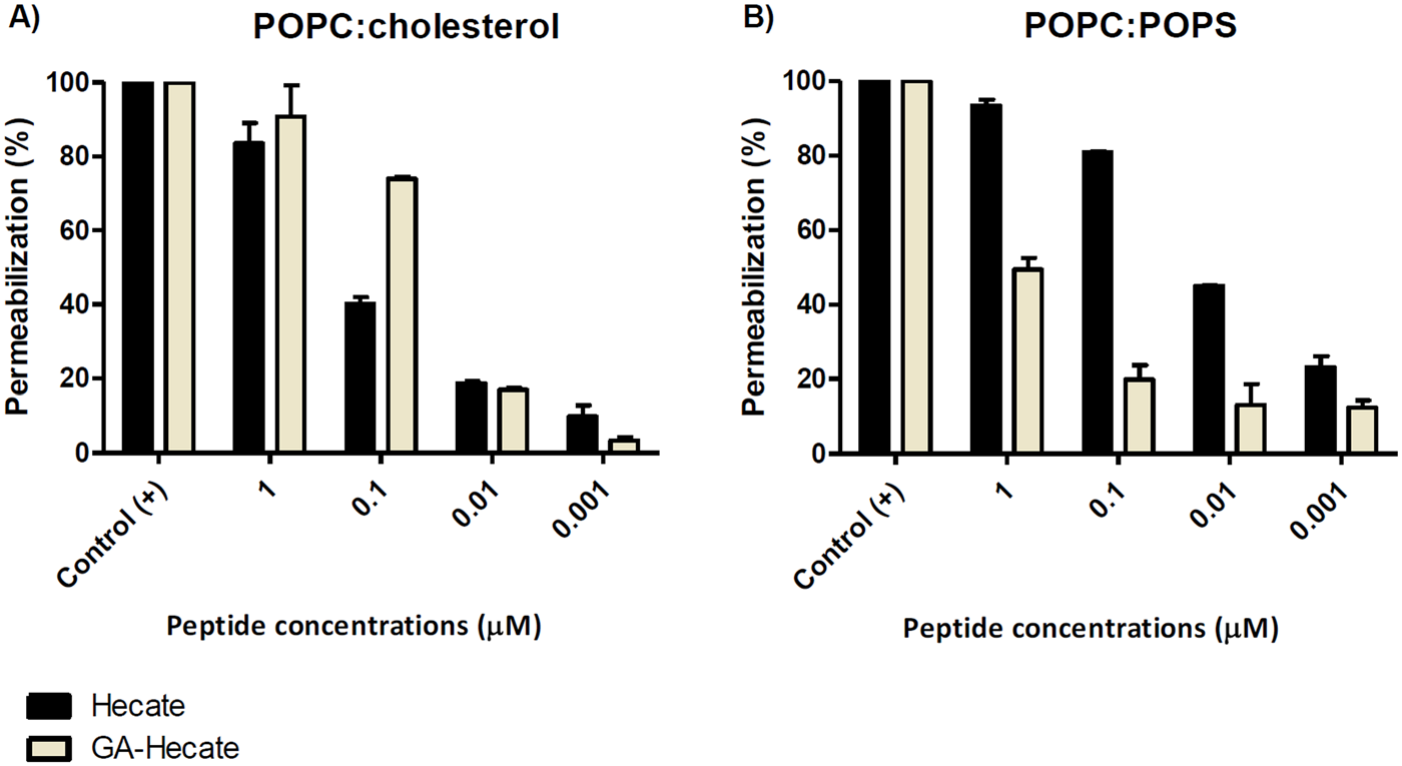
Permeabilization of LUVs. **(A**) Percentage of carboxyfluorescein release in POPC:POPS (9:1) Post addition of LUVs at different concentrations of Hecate and GA-Hecate. (**B**) Percentage of carboxyfluorescein release results in POPC:Cholesterol (9:1) LUVs after the addition of different concentrations of Hecate and GA-Hecate. The bars indicate SD.

To confirm the biologic results of GA-Hecate’s lipid interaction, we used mimetic unilamellar vesicles containing lipid droplet or envelope/cellular membrane constitution. GA-Hecate showed a direct interaction with the large unilamellar vesicles (LUVs) with a similar constitution of lipid droplets (POPC: Cholesterol; 9:1; m:m), permeabilizing approximately 70% of these LUVs when added at 0.1 μM. This effect was approximately 2-fold higher than the Hecate effect (Fig. 8A). GA-Hecate was also able to interact with LUVs with a similar constitution of viral envelope and host cell membrane (POPC:POPS; 9:1; m:m). However, this interaction was not as strong as that presented for lipid droplet mimetics, and the same concentration of GA-Hecate presented about a 20% permeabilization capacity. GA-Hecate interaction capacity for POPC:POPS LUVs ranged from 2- to 3-fold lower than Hecate interaction capacity (Fig. 8B).

## Discussion

Hecate is a lytic peptide that presents a well-established capacity to interact with lipids, intercalating in lipid bilayers and generating disruption^10^. The viral envelope is derived from the host cell lipid bilayers and therefore is constituted by host cell lipids^16^ associated with viral envelope proteins. Thus, Hecate’s capacity to interact with lipids could alter the membrane fluidity, disturb viral envelope formation, and inhibit both virus fusion and egress. In addition, inside the cell, it could interact with *lipid droplets*, which are small lipid organelles used by HCV during its replication and assembly and are responsible for transportation of HCV proteins and replicative complexes between cell compartments^14,17^.

This study showed Hecate’s capacity to inhibit HCV replication in a dose-dependent manner, while also presenting high toxicity on hepatocytes. To further exploit the inhibitory capacity of Hecate on HCV, we introduced some changes in its N-terminus region, which is associated with the modification of biological properties of Hecate^9,18,19^. In this context and to assess the relationship between structure and biological activity, the general positive charge of Hecate was increased or reduced by addition of positive (Lysine - Lys) or negative (Glutamic acid - Glu) amino acid residues, respectively, or by the introduction of non-amino acid radicals (acetyl or Gallic acid). These modifications were performed aiming to keep the antiviral activity and decrease its lytic properties on the host cells.

The addition of a positive amino acid residue (Lys) to Hecate (Lys-Hecate) increased its antiviral potency as well as its cytotoxicity in hepatocytes. The interaction of negatively charged biomembrane components, mostly phospholipids, with peptide cationic domains is suggested to be the main mode of action of the antimicrobial peptides^20^. Thus, we proposed that this increase in the effects of the peptide could indicate a higher ionic interaction with lipids and consequently a higher destabilization in both virus and cell membrane lipids arising from a major charge difference.

To confirm this property, we reduced the general charge of Hecate. The peptides GA-Hecate and GA_2_-Hecate reduced both the peptide potency against HCV and hepatocyte toxicity; however, the same was not true for the other, less positive peptides, acetyl and Glu-Hecate, which increased both the toxicity and anti-HCV effect. Thus, we raise the possibility of steric hindrance as the determining factor in this case. Steric hindrance is a well-established property in peptide chains arising from many atoms that share the same space^21^. The increased steric effect presented by GA-Hecate and GA_2_-Hecate due to the larger Gallic acid structure had less effective interactions with membrane lipids in the host cell, causing less cytotoxicity. The same effect could be found on the lipid droplets inside the cells, causing a reduced effect on replication. Small molecular groups present have a lower geometric proximity between atoms and a consequently lower steric hindrance^21^. Thus a lower effect of GA-Hecate compared to GA_2_-Hecate favoured the GA-Hecate balance between toxicity and HCV inhibition. The reduction in the positive charge presented by the acetyl- and Glu-Hecate peptides was not enough to avoid interactions with negative membrane charges and the effect presented by acetyl- and Glu-Hecate could be explained by secondary structure changes.

Changes in the secondary structure were already described in GA-Hecate, which presented a reduced α-helix in negative membranes compared to Hecate. This structural change has been associated with the reduced toxicity of GA-Hecate in tumor and non-tumor cells^9^, property which was confirmed in this study in hepatocytes. Together, our results show that the effects on the host cell and viral replication are triggered by the same properties until a threshold.

HCV is a highly diverse virus that is divided into genotypes, which have differences in their global distribution and treatment responsiveness. Genotype 1 is the most prevalent in the world, followed by genotype 3^22^ and, due to its low responsiveness in the initial anti-HCV treatments, genotype 1 has been the main target of research in last decades. However, recent years highlight the importance of multi-genotype antivirals. Thus, we evaluate the Hecate spectrum on HCV. All initial peptides were also tested for HCV genotype 3a and showed similar results to genotype 2a. However, it is important to note that most of the peptides, except GA-Hecate, were more toxic in Huh-7.5 cells harbouring genotype 3, which could be related to its more severe effects on hepatocytes^23^, making these cells more sensitive to any sort of stress. GA-Hecate was also tested for HCV genotype 1b and showed high efficiency of inhibition but was less efficient on genotype 1b when compared to the other genotypes.

Due to its more efficient spectrum of action and potency, GA-Hecate was tested on other viral replication steps. GA-Hecate inhibited approximately 85% of the replication of HCV cell culture-derived viral particles (HCVcc) at 20 μM in a dose-dependent manner. At the same concentration, GA-Hecate also showed a high efficiency against viral entry inhibition, reducing approximately 95% of focus forming units in a dose-dependent manner. The effect on HCV release was moderate and was not dose-dependent, showing approximately 50% of inhibition for all safe concentrations (≥20 μM). However, when the particles released from GA-Hecate-treated cells were used to infect naïve Huh-7.5 cells, they were not able to generate a new infection, showing that even though GA-Hecate only partially blocks viral egress, it generates impaired viral particles. These results strengthen the possibility of lipid action once approximately 50% of HCV RNA is detected in the supernatant, but less than 1% can generate a new infection. Additionally, supporting the capacity of GA-Hecate to interact with lipids, its main structure (Hecate) was shown to inhibit the Herpes simplex virus type 1 cell fusion and virus egress, which was related to changes in membrane fluidity^24^.

Intercalation in the membrane lipids is the most well-established property of Hecate^8,10^ and, although it is primarily associated with its charge, we analysed the GA-Hecate capacity to affect neutral lipids commonly used by HCV both in replication and particle assembly. GA-Hecate reduced and redistributed the total content of the cytosolic lipid droplets (LD), moving the droplets away from the nuclei and presenting a scattering pattern through the cytoplasm, but not changing the LD size. Lipid droplets have been associated with the core-dependent transport of the viral proteins, as well as the HCV replication complexes through the cell^14^. Perinuclear localization of the lipid droplets is essential for the HCV replication process, once the envelope proteins reside in endoplasmic reticulum (ER) lumen, and the viral replicase is assumed to be localized on ER-derived membranes^25^. Confirming the biological results, GA-Hecate had a direct interaction with the large unilamellar vesicles similar to the lipid droplets (POPC:cholesterol). This peptide also showed a high efficiency to interact with LUVs, presenting a similar constitution of the virus envelope and host cell membrane (POPC:POPS), but this interaction was weaker than that presented for the lipid droplet mimetics. The interaction with POPC:POPS could be explained by the charge difference among peptides and the LUVs. Membranes containing POPS have a negative charge, while the tested peptides present net positive charges. Both compounds interact with these negative membranes, but GA-Hecate, which presents less positive charge, showed less interaction and activity in the negative vesicles in relation to Hecate. This property can also be linked to a lower interaction with the virus envelope and the host cell membrane. POPC:Cholesterol membranes present a neutral charge, similar to the lipid droplets. Thus, the influence of peptide charge cannot be considered for the lipid droplet interaction, which also confirms the biological findings. The CD spectroscopy of Hecate and GA-Hecate peptides in the lipid vesicles showed that the addition of GA at the N-terminal position decreased the α-helix percentage of the peptide in the negatively charged membranes (see Supplementary Figs S6, S7). In uncharged membranes, Hecate does not show a clear secondary structure formation. However, GA-Hecate showed a mixture of random coil and α-helix structures. This higher structuration of the derivative in the neutral vesicles could be related to the lipid droplet activity in the permeabilization studies.

Sanches *et al*.^9^ proposed that aggregation of many GA-Hecate molecules in the membranes is necessary to generate its disruption, and that in low concentrations, this peptide could have the capacity to cross biomembranes and act on components inside the cells without disrupting the cell membrane. This property is supported by Hecate’s capacity of keeping its effect on treated cells, even though it is removed after some hours of treatment (data not shown)^24^. We here confirmed a membrane disruption triggered by GA-Hecate accumulation, since at high concentrations it induces necrosis, but this type of death was not generated in concentrations lower than 20 µM. On the other hand, no pro-apoptotic effect is showed in any dose or time until 72 h post-treatment. The ability of GA-Hecate to keep its viral inhibition ability in very low concentrations face to no toxicity or apoptosis induction, reinforce the balance between virus inhibition and host cell survival produced by GA-Hecate. Thus, due to the aggregation necessity and a much higher area of cells in comparison to the virus, GA-Hecate may be capable of generating disruption of the viral envelope, but not of the host cell lipid bilayer. In addition, we believe that the main function of the peptide is performed inside the cell, changing the membrane fluidity and interacting with the intracellular lipids. Considering the GA-Hecate capacity to interact more efficiently with the neutral mimetic vesicles, this capacity to cross the membrane also explains a direct interaction with the lipid droplets which is surrounded by a phospholipid monolayer^14^ and thus is easier to disrupt.

HCV is a single-stranded, positive-sense RNA ((+)ssRNA) virus, which generates a negative sense intermediate during its replication as template for new positive sense RNA production^26^. During this period, a double-stranded RNA is generated as an intermediate in genome replication for positive sense ssRNA virus^27^. Considering that some cationic peptides have the capacity to intercalate in dsRNA via ionic interactions^28^, we also evaluated this capacity for GA-Hecate (charge +9) and compared it with Hecate (charge +10) and Lys Hecate (charge +11) (see Supplementary Fig. S8)^29^. All tested peptides showed the same efficiency to intercalate in HCV dsRNA, which indicated that the charge change does not influence the intercalation capacity. These data showed that the peptide/lipid interaction is more crucial for the viral replication effect than the intercalating property, which is consistent with similar inhibition behaviours on the host cells and virus. The property of intercalating in dsRNA, as an ionic interaction, allows for the possibility that GA-Hecate could interact with dsRNA from other single-stranded RNA viruses, and thus could also inhibit these viruses. This property presented by GA-Hecate and Hecate, as well as the ability to interact with lipids suggests that these peptides are at least interesting candidates for further research as wide spectrum antivirals.

In conclusion, GA-Hecate inhibits major steps in the HCV replication cycle, which represents a more efficient infection control. GA-Hecate’s main effect is predicted to be inside the cells, scattering the lipid droplets and intercalating in the dsRNA replication intermediary but it also could change the membrane fluidity. GA-Hecate could guide the development of antiviral drugs, representing an efficient and easily diffusible treatment for HCV and possibly for other viruses but also as a tool to clarify lipid dynamics between the host cells and viruses with a lipid-dependent cycle as in HCV.

## Material and Methods

### Peptide synthesis

The synthesis of peptides was manually performed via solid-phase peptide synthesis (SPPS) using the standard Fmoc (9-fluorenylmethyloxycarbonyl) protocol on a Rink-MBHA resin. The identity of the peptides was confirmed by electrospray mass spectrometry, and the final purity of the peptides was higher than 95%^9^.

### Antibodies and reagents

Anti-NS3 and anti- mouse polyclonal antibodies were purchased from Abcam (San Francisco, CA, USA), and anti-NS5A was kindly provided by Professor Mark Harris (University of Leeds, UK). Bodipy 493/503 was purchased from Thermo-Scientific (Pierce, Rockford, IL, USA), 4′,6-diamidino-2-phenylindole (DAPI), paraformaldehyde and MTT were purchased from Sigma-Aldrich (St. Louis, MO, USA).

### Cells and virus

The human hepatocellular carcinoma cell line (Huh7.5) was cultured in Dulbecco’s modified Eagle’s medium (DMEM - Gibco - Life Technologies, USA) supplemented with 10% fetal bovine serum (Cutilab, Campinas, SP, BR), 100 IU/mL penicillin, 100 μg/mL streptomycin, 1% (v/v) non-essential amino acids (Gibco - Life Technologies, USA) and maintained at 37 °C in a humidified 5% CO_2_ incubator. Cell lines harboring HCV subgenomic replicons (SGR), SGR-Feo-JFH-1 (genotype 2a^30^) and S52/SG-neo (genotype 3a^31^) were maintained in DMEM supplemented additionally with 500 μg/mL G418 (Sigma-Aldrich). The HCV full length replicons used were FL-J6/JFH-50C19Rluc2AUbi^32^ and JFH-1^33^, both genotype 2a. Huh-7.5 cells harboring HCV full length replicons were maintained in DMEM supplemented additionally with HEPES 20 mM (Sigma-Aldrich).

### The cytotoxicity profile of Hecate and its derivatives in hepatocytes

Peptide cytotoxicity profiles were measured by the MTT [3-(4,5-dimethylthiazol- 2-yl)-2,5-diphenyl tetrazolium bromide] (Sigma–Aldrich) method. Twenty-four hours before treatment, 5 × 10^3^ SGR-harboring cell lines (SGR-Feo-JFH-1 or S52/SG-neo) were cultured with supplemented DMEM in a 96-multi-well plate and incubated at 37 °C in a humidified 5% CO_2_ incubator. On the subsequent day, peptide-containing medium was added at the following final concentrations: 2.5, 5, 10 and 20 μM. The tested peptides were Hecate and its derivatives, which included the following: lysine addition (Lysine-Hecate/Lys-Hecate); one or two molecules of Gallic Acid (GA-Hecate and GA_2_-Hecate); one acetyl radical (Acetyl-Hecate) or Glutamic acid (Glu-Hecate). After 48 h of incubation, peptide-media was removed, and MTT at a final concentration of 1 mg/mL was added to the wells, incubated for 30 minutes and subsequently replaced by 100 μL of DMSO. Absorbance was measured at 562 nm, using a plate spectrophotometer (FLUOstar Omega/BMG LABTECH, Offenburg, BW, DE). Cell viability was calculated considering water-treated cells (peptides diluent) as a negative control. All experiments were performed in triplicate and in three independent replicates. Further assays were performed considering at least 80% viability of the treated cells.

### HCV replication assessment under treatment with Hecate or its derivative

Cell lines harboring subgenomic or genomic replicons were seeded at 5 × 10^3^ cells /well on 96-well plates and the compounds were added 24 h post-seeding, in a concentration range between 2.5–20 μM. After 48 h of treatment, the cells were disrupted with 30 μL of passive lysis buffer (Promega, Madison, WI, USA), and luciferase substrate (Promega, Madison, WI, USA) was automatically added to each sample. Luciferase activity was measured using a luminometer (FLUOstar Omega/BMG LABTECH, Offenburg, BW, DE). The same assays were performed in Huh-7.5 cell lines harboring SGR-JFH1 Feo, S52/SG-neo or FL-J6/JFH-50C19Rluc2AUbi. Cyclosporine A (Sigma-Aldrich) at 1 μM was used as positive control of viral replication inhibition and dH_2_O as negative control.

### Western blotting

HCV (FL J6/JFH) infected cells were used to evaluate virus protein expression after peptide treatment. Twenty-four hours before treatment, 2 × 10^5^ infected cells were seeded in 6 well-plates and on the subsequent day were treated with GA-Hecate (5, 10 and 20 μM) for 48 h and then lysed with CellLytic^TM^ M (Sigma-Aldrich) containing protease inhibitors (Sigma-Aldrich). Cyclosporine A was used as a positive control of inhibition and dH_2_O as a negative control of inhibition. Total protein was quantified using a BCA assay (Thermo-Scientific) following the manufacturer’s instructions. Subsequently, the proteins were separated by SDS/PAGE, transferred to a PVDF membrane (Millipore, Bedford, MA, USA), blocked in TBS-T/5% skimmed milk (Bio-RAD, Amadora, PT) and incubated overnight with a polyclonal anti-NS3 mouse antibody (1:3,000) (Abcam, San Francisco, CA, USA). The membrane was then incubated with anti-mouse IgG (whole molecule) (Abcam, San Francisco, CA, USA) for 1 h. Finally, the membrane was incubated for 1 minute with Pierce ECL Western Blotting Substrate (Thermo-Scientific-Pierce, Rockford, IL, USA), and the luminescence intensity was captured on a Chemi-Doc System (Bio-Rad, Amadora, PT). GAPDH (Abcam, San Francisco, CA, USA) was used as the endogenous control for the normalization of protein expression.

### Release/Assembly evaluation

JFH-1-infected Huh-7.5 cells were used to evaluate the GA-Hecate effect on virus release/assembly. Forty-eight hours prior to treatment, 2 × 10^5^ cells harboring JFH-1 were seeded on 6 well-plate, and 48 h post-seeding GA-Hecate was added (5, 10 and 20 μM) following the previously described protocol^34^. After treatment, the intracellular and extracellular RNA content was isolated following the TRIzol reagent protocol and quantified by qPCR of the HCV 5′ UTR region (forward: 5′ CGGGAGAGCCATAGTGG; reverse, 5′ AGTACCAACAAGGCCTTTCG; probe: 5′-FAM-CTGCGGAACCGGTGAGTACAC-TAMRA-3) using TaqMan® Universal PCR Master Mix (Thermo Scientific, CA, USA). The difference between the intra/extracellular expressions showed the HCV release behavior after treatment for 24 h with GA-Hecate.

### Infectivity assay for GA-Hecate

To assess the effect of GA-Hecate on HCV virion infectivity, a similar process to release/assembly was done and the supernatant (extracellular content) was collected and used to infect naïve Huh-7.5 cells. HCV infectivity was evaluated by the focus forming units assay, following an indirect immunofluorescence (IFI) assay for HCV NS5A as previously described by *Batista et al*.^35^.

### GA-Hecate effect on entry step

To evaluate the GA-Hecate capacity to block the HCV entry step, 5 × 10^3^ Huh-7.5 cells were seeded 24 h before infection. On the following day, the supernatant at a multiplicity of infection (M.O.I) of 0.1 was inoculated in the cells concomitantly (co-treatment) with GA-Hecate (5, 10 and 20 μM). The treatment was kept for 4 hours and then removed. Additionally, virucidal analysis and pretreatment analysis were performed. For virucidal analysis virus was incubated with the drug during 1 h at 37 °C prior to the cells infection and for pretreatment, cells were incubated with the drug during 1 h at 37 °C and subsequently infected. Cells were maintained for further 72 h. At the end of the treatment, the cells were summited to an indirect immunofluorescence assay following the focus forming unit method using IFI.

### Intracellular distribution and quantity of neutral lipids

To evaluate the GA-Hecate effect on the intracellular neutral lipid distribution, the GA-Hecate-treated cells at maximum non-cytotoxic concentration (20 μM) were evaluated 48 h post-treatment. For this purpose, 2 × 10^4^ Huh-7.5 cells that were infected with HCVcc were seeded in a 24-well plate 24 h following the treatment. On the following day, GA-Hecate or water (peptide diluent) were added in the plate and incubated for 48 h. At the end of treatment, the cells were fixed using 4% paraformaldehyde, submitted to IFI assay for NS5A detection and stained by Bodipy 493/503 (Thermo Fischer) at a final concentration 1 μg/mL for 10 minutes. The nucleus was stained using DAPI for 5 minutes. To quantify the intracellular lipid content, two - four photos/well were performed and the fluorescence intensity was measured by ImageJ software. Bodipy fluorescence was normalized with DAPI fluorescence.

### GA-Hecate interference in lipid droplet size

To determine the GA-Hecate capacity for changing the lipid droplet size, we performed a light scattering dynamic assay. For this purpose, we used large unilamellar vesicles that were composed of phosphatidylcholine and Cholesterol (POPC:Cholesterol; 9:1; m:m), simulating the general constitution of lipid droplets that were synthetized in phosphate buffered saline (PBS) at pH 7.2. The vesicle diameter was measured between 0.1 and 1,000 nm before and after peptide treatment. The peptide tested concentrations ranged from 0.5 to 0.05 μg/mL, representing the technique threshold. PBS was used as a negative control of size change and 1% Triton-X 100 was used as a positive control of size change. The assay was performed in triplicate in a single assay.

### Hecate’s and GA-Hecate’s permeabilization effect on the mimetics of lipid droplets and viral envelope

Phosphatidylcholine:Cholesterol (POPC:Cholesterol - 9:1; m:m) and phosphatidylcholine:phosphatidylserine (POPC:POPS - 9:1; m:m) vesicles, which mimic lipid droplets and the virus envelope, respectively, were prepared at a concentration of 15 mmol·L^−1^. Each mixture was submitted to lipid solubilization in chloroform/methanol (4:1; v:v) and subsequent evaporation by nitrogen gas flow, resulting in the formation of a lipid film. The product evaporated using a vacuum procedure for complete removal of the solvents and then a solution containing the fluorophore carboxyfluorescein (CF; 50 mM) was added. The mixture was warmed up to 60 °C, sonicated and agitated for equal periods for 10 min to allow for the formation of vesicles encapsulated with CF. Following this, the mixture was processed in an extruder machine (Avanti Polar Lipids) to obtain large unilamellar vesicles. The obtained solution was then applied to a size exclusion column with Sephadex G20 resin for separation of the unencapsulated CF. The action of Hecate and GA-Hecate on these vesicles was accompanied by the release of the fluorophore in a spectrofluorometer (Cary Eclipse Varian) set at 492 nm and 517 nm for excitation and emission, respectively. A solution of 1% Triton in water was used as a positive control (100% permeabilization) and milliQ water was the negative control for permeabilization.

### Statistical analysis

All statistical analyses, except the release assays, were performed by one-way ANOVA with Tukey’s post-test. Release assays were analysed by two-way ANOVA with Bonferroni post-test. Viability, replication, entry and release assays were performed in triplicate in three independent events (n = 9). Western blotting assays were performed twice. Infectivity assays were performed in three repetitions (n = 3). Intercalation assays and lipid distribution assays were performed twice (n = 2). Light scattering assays and permeabilization assays were performed once in triplicate (n = 3). All statistical tests were performed using GraphPad Prism 5.0 software (GraphPad Software, San Diego, CA, USA).

## Electronic supplementary material

Supplementary Material

## Data Availability

All data generated or analysed during this study are included in this published article (and its supplementary information files).
