# Supplementary material for: GA-Hecate antiviral properties on HCV whole cycle represent a new antiviral class and open the door for the development of broad spectrum antivirals
Source: Sci Rep. 2018 Sep 25;8:14329. doi: 10.1038/s41598-018-32176-w (PMC6156508; doi:10.1038/s41598-018-32176-w)
Supplement: Supplementary file 1 — Supplementary Material [file 41598_2018_32176_MOESM1_ESM.pdf]

Supplementary Material For

GA-Hecate antiviral properties on HCV whole cycle represents a new antiviral class and opens the door for the development of broad spectrum antivirals

Mariana Nogueira Batista[1+]; Paulo Ricardo da Silva Sanches[2+] ; Bruno Moreira Carneiro[1]; Ana Cláudia Silva Braga[1]; Guilherme Rodrigues Fernandes Campos[1]; Eduardo Maffud Cilli[2]; Paula Rahal.[1*]

[1]Institute of Bioscience, Language and Exact Science, UNESP - São Paulo State University, São José do Rio Preto, SP, Brazil.

[2]Institute of Chemistry, UNESP – São Paulo State University, Araraquara, SP, Brazil.

[+]Authors contributed equally to this work

*Corresponding authors: prahal@ibilce.unesp.br ; cilli@iq.unesp.br

GA-Hecate toxicity and viral inhibition profile in 24, 48 and 72h post-treatment in genotype 2a

GA-Hecate toxicity and viral inhibition capacity were evaluated at 24, 48 and 72h post-treatment. Tested concentrations were 1.25; 2.5; 5; 10; 20; and 40 µM. At the end of every incubation, cells were stained using MTT as previously described. Similarly, at every time point, treated cells were lysed using PLB and summited to Luminescence analysis as previously described.

Apoptosis and Necrosis analysis

The ability of GA-Hecate inducing cell death by necrosis and apoptosis was assessed in 48 and 72h post-treatment (p.t) at concentrations: 1.25; 2.5; 5; 10; 20 and 40 µM. For necrosis evaluation, cells were stained using Propidium Iodide at 1M, which was incubated with the cells 15 minutes prior fixation. Subsequently, cells were fixed using paraformaldehyde 4% in PBS (PFA 4%) for 15 minutes and nuclei stained using DAPI 1 µg/mL for 5 minutes. For apoptosis evaluation, cells were fixed using PFA 4% during 15 minutes, permeabilized using Triton X- 100 (Sigma) 0.1% in PBS and stained using anti-Active Caspase 3 antibody (host: Rabbit) (1:200 in BSA 1% in PBS) (Abcam 13847) for 2h. Then cells were rinsed using PBS and stained using anti-rabbit AlexaFluor 594 (1:500 in BSA 1% PBS) (Thermo Scientific A21207) during 2h. Nuclei were stained using DAPI for 5 minutes. For both assays, cells were counted in 5 randomly chosen fields in duplicate.

GA-Hecate toxicity and viral inhibition profile in HCV genotype 1b

In order to evaluated GA-Hecate effects on HCV genotype 1b, $2x10^6$ cells Huh-7.5 were electroporated using 2µg of SGR- BM45-Feo (genotype 1b). Subsequently, $5x10^3$ cells were seeded into 96-well plate and after 24h, GA-Hecate was added at the following concentrations: 1.25; 2.5; 5; 10; 20; and 40 µM. GA-Hecate toxicity and viral inhibition capacity were evaluated 48h post-treatment by MTT and by Luciferase Reporter Assay.

Circular Dichroism

Circular dichroism (CD) spectra were obtained between 190 and 260 nm with a JASCO J-815 CD spectrophotometer (Japan) on nitrogen flush in 1 mm path-length quartz cuvettes at room temperature. All CD spectra were acquired using an average of 8

scans, with a resolution of 0.2 nm. To investigate the conformational changes by the membrane environments, the data were obtained in vesicles containing POPC:POPS (9:1), and POPC:Cholesterol (9:1). CD spectra were typically recorded as an average of the eight scans that were obtained in millidegrees and converted to molar ellipticity [θ] in $deg \cdot cm^2 \cdot dmol^{-1}$. The peptide concentration was 10 $\mu mol \cdot L^{-1}$, and the proportion of lipid/peptide was 100 (L/P = 100).

Effect of peptides on dsRNA intercalation assay

To assess GA-Hecate's dsRNA intercalation capacity, we performed an assay adapted from Krawczyk *et al.* 2009. Initially, a PCR reaction was made and the HCV JFH1 3' untranslated region (UTR) (accession no. AB047639) was amplified by specific primers flanked by a T7 promoter sequence (F TAATACGACTCACTATAGGGGGCACACACTAGGTACA; R-TAATACGACTCACTATAGGGACATGATCTGCAGAGAG). The amplicon, with 273 bp, was purified using a Zymoclean™ Gel DNA recovery Kit (Zymo Research) and used as a template for *in vitro* dsRNA production using a T7 Ribomax Express kit (Promega). The double-stranded molecule was obtained by complementary annealing obtained by temperature reduction. GA-Hecate, Hecate and Lys-Hecate (20 µM) were incubated with 15 mM of dsRNA, for 45 min, and subsequently analysed in 1% agarose gel 1X TAE stained with ethidium bromide. As a positive control, we used Doxorubicin (100 µM).

Results

Cell viability

The peptide showed an inhibition time and dose dependent. The maximum effect with lower cell death was reached at 48h (Figure S1). The maximum toxicity is reduced at 72h and is reached at 10 µM. An increased ability to inhibit the virus in lower concentrations is reached at 72h post-infection and the lower used concentration (1.25 µM) inhibited about 50% of the viral replication and presented no cell toxicity. IC50 and CC50 of GA-Hecate at this time point were 3.8 µM and 19 µM respectively (SI: 5). In order to guarantee that the virus inhibition effect was not related exclusively to cell death trigger, all concentrations were evaluated to necrosis and apoptosis induction.

Necrosis and Apoptosis

At 40 μM, GA-Hecate induced 50% of necrosis 48h p.t. However, at 72h p.t this percentage is reduced to 10%, considering that the cells which were in necrosis process at 48h are already dead at 72h p.t. and the increase in the cell death presented by MTT assays was attributed to the end of the necrosis process at 72h (Figure S2 and S3). The 20% of death found at 20 μM was also attributed to necrosis, since there was no significate apoptosis induction in any time point for this concentration and the percentage of necrosis at this concentration is about 20% over the time (Figure S2 and S3). The peptide showed nonselective properties at high concentrations. However in low concentrations there was selectivity for virus interaction over cell interaction as depicted in the figure S4.

GA-Hecate effect on HCV genotype 1b subgenomic Replicon

GA-Hecate presented a reduced ability to inhibit genotype 1b replication when compared to the other tested HCV genotypes and reached a maximum inhibition of 65% in the maximum safe concentration.

GA-Hecate intercalation property in dsRNA

To further evaluate the influence of charge on peptide effect and considering the property of some peptides to intercalate into dsRNA, we also evaluated this capacity for GA-Hecate (charge +9) and compared it with Hecate (charge +10) and Lys-Hecate (charge +11). All tested peptides were able to intercalate in HCV dsRNA (Figure S8).

GA-Hecate and Hecate structuration in negatively charged and uncharged membranes

CD spectroscopy of Hecate and GA-Hecate peptides in lipid vesicles showed that the addition of GA at the N-terminal position decreased the α-helix percentage of the peptide in negatively charged membranes. In uncharged membranes, Hecate does not show a clear secondary structure formation (Fig. S1). However, GA-Hecate showed a mixture of random coil and α-helix structures (Fig. S2).

Supplementary Figures

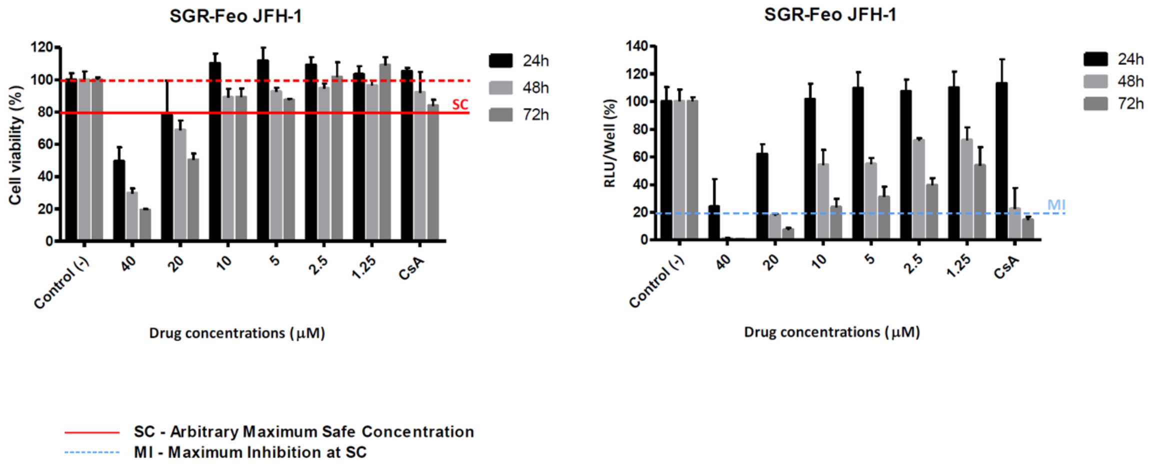

Figure S1. **Viability and Replication assessment of GA-Hecate using SGR-Feo JFH-1 at different incubation periods**. A) Cell Viability assessed in 24, 48 and 72h post-treatment. Water was used as negative control for cell death. B) Replication inhibition presented by GA-Hecate in 24, 48 and 72h post-treatment.

A)

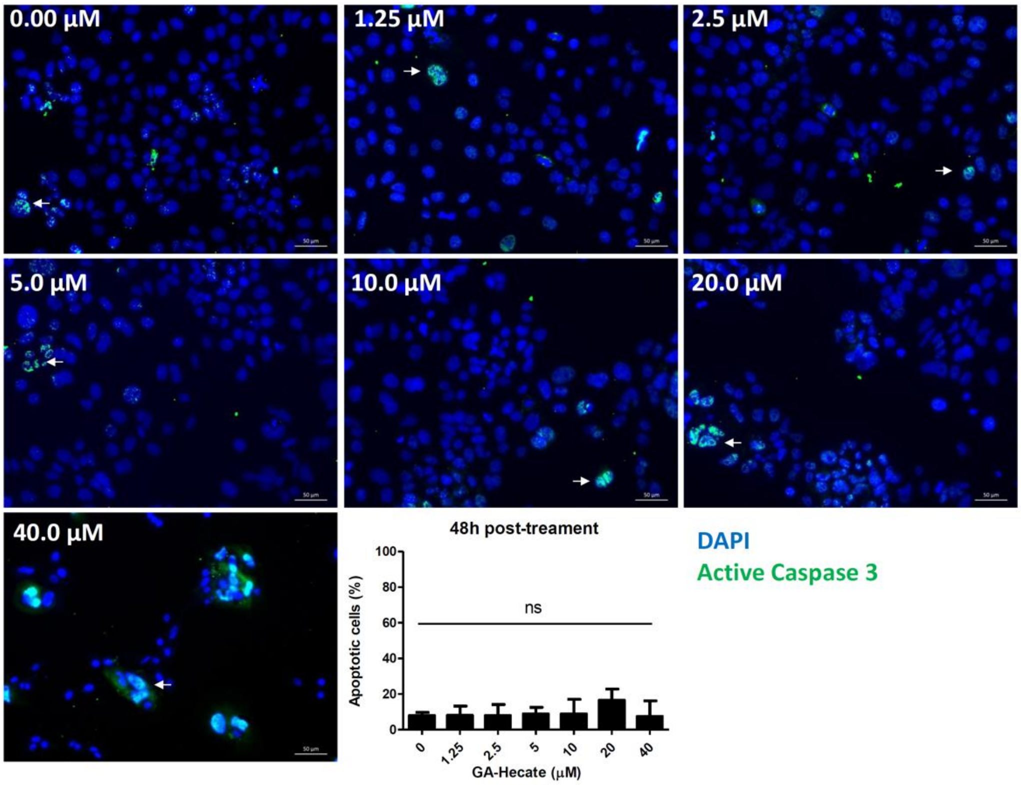
0.00 µM
1.25 µM
2.5 µM
5.0 µM
10.0 µM
20.0 µM
40.0 µM
48h post-treament
Apoptotic cells (%)
ns
GA-Hecate (µM)
0
1.25
2.5
5
10
20
40
DAPI
Active Caspase 3

B)

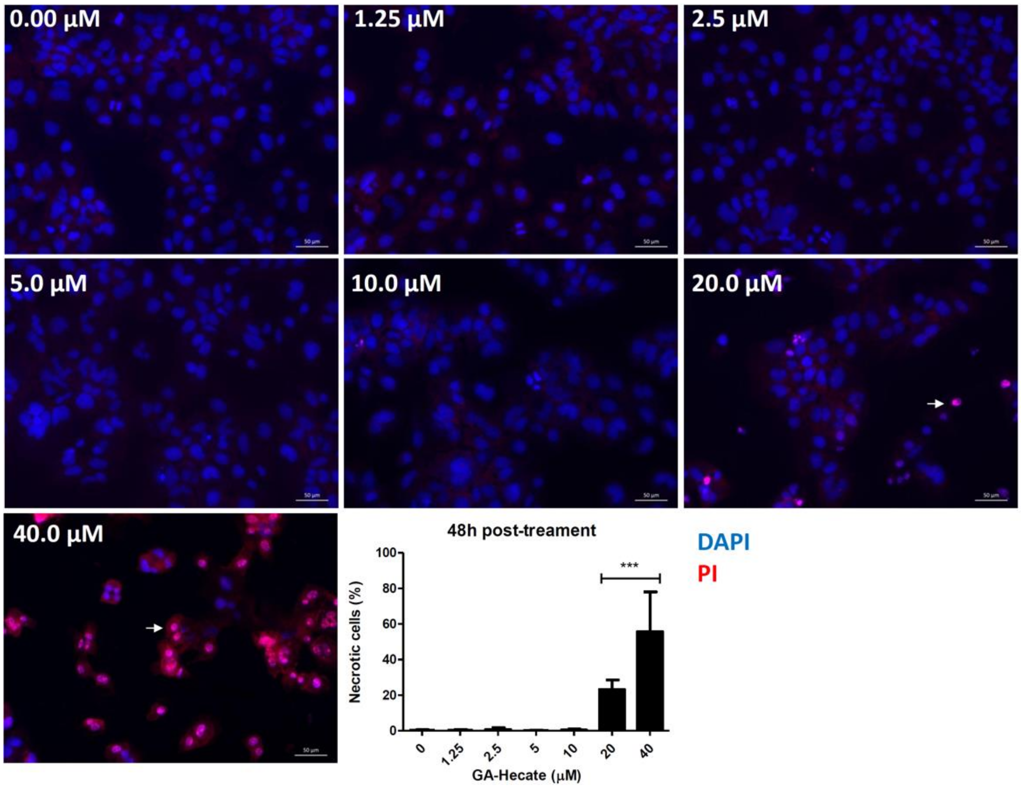
0.00 µM
1.25 µM
2.5 µM
5.0 µM
10.0 µM
20.0 µM
40.0 µM
48h post-treament
Necrotic cells (%)
***
GA-Hecate (µM)
0
1.25
2.5
5
10
20
40
DAPI
PI

Figure S2. **Cell death mechanisms triggered by GA-Hecate 48h post-treatment in J6/JFH-1- infected Huh-7.5 cells.** A) Apoptosis activation triggered by GA-Hecate was measured by Active Caspase-3 in treated cells. Caspase is artificially represented in green and nuclei were stained using DAPI 1µg/mL (blue). B) Necrosis assessment. Necrotic cells were stained using Propidium iodide 1M, which is able to cross only damaged membranes and reaches the nucleus. PI is represented in red and cell nuclei were counterstained using DAPI (blue). Bars represents the average of 5 fields in duplicate. Ns: non statistically significant. *** Pvalue< 0.001vs 0.00 µM

A)

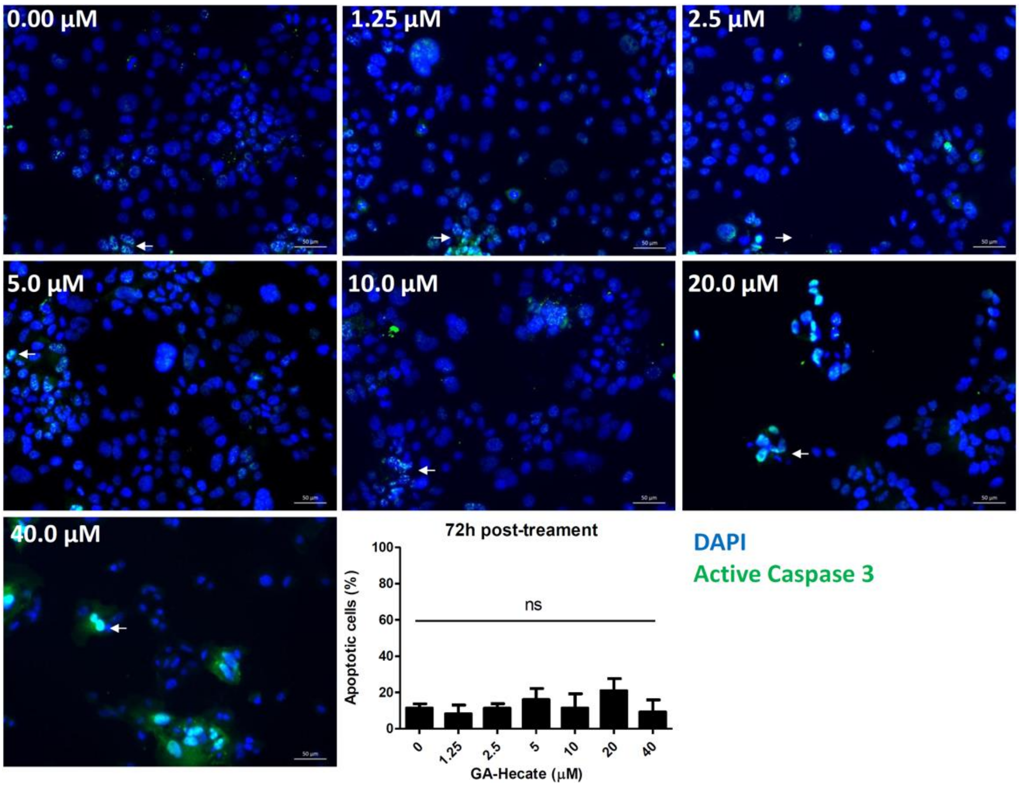
0.00 µM
1.25 µM
2.5 µM
5.0 µM
10.0 µM
20.0 µM
40.0 µM
72h post-treament
Apoptotic cells (%)
100
80
60
40
20
0
ns
0
1.25
2.5
5
10
20
40
GA-Hecate (µM)
DAPI
Active Caspase 3

B)

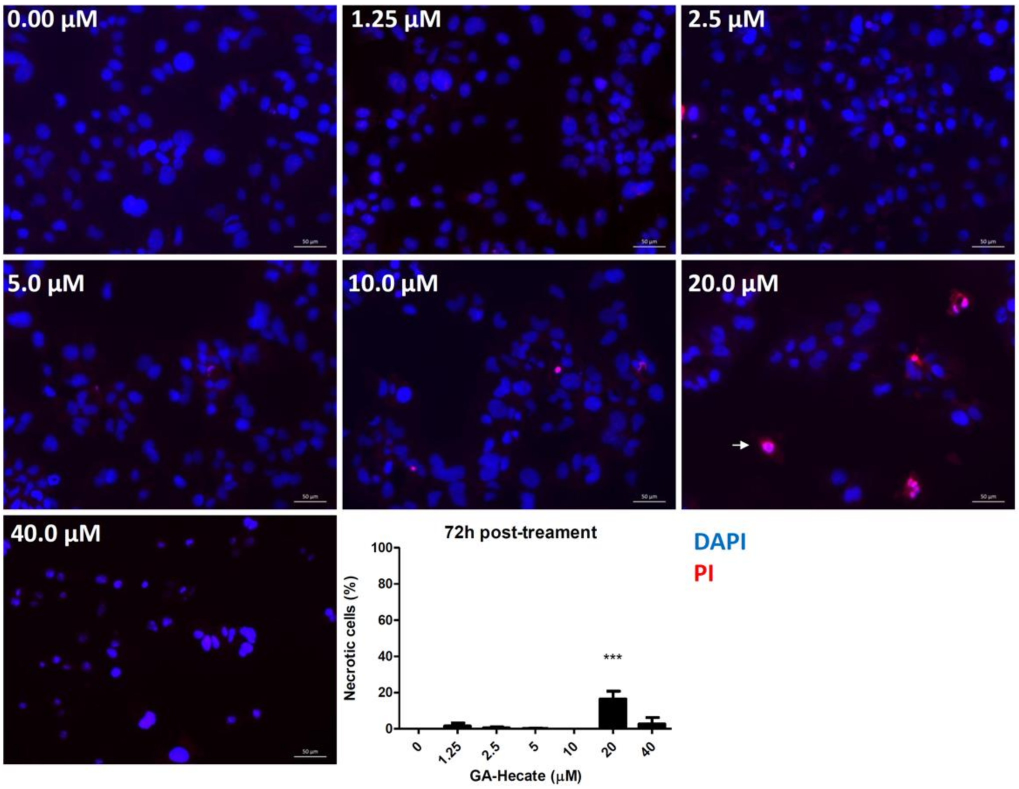
0.00 µM
1.25 µM
2.5 µM
5.0 µM
10.0 µM
20.0 µM
40.0 µM
72h post-treament
Necrotic cells (%)
100
80
60
40
20
0
***
0
1.25
2.5
5
10
20
40
GA-Hecate (µM)
DAPI
PI

Figure S3. **Cell death mechanisms triggered by GA-Hecate 72h post-treatment in J6/JFH-1- infected Huh-7.5 cells**. A) Apoptosis activation triggered by GA-Hecate was measured by Active Caspase-3 in treated cells. Caspase is artificially represented in green and nuclei were stained using DAPI 1µg/mL (blue). B) Necrosis assessment. Necrotic cells were stained using Propidium iodide 1M, which is able to cross only damaged membranes and reaches the nucleus. PI is represented in red and cell nuclei were counterstained using DAPI (blue). Bars represents the average of 5 fields in duplicate. Ns: non statistically significant. *** Pvalue< 0.001vs 0.00 µM

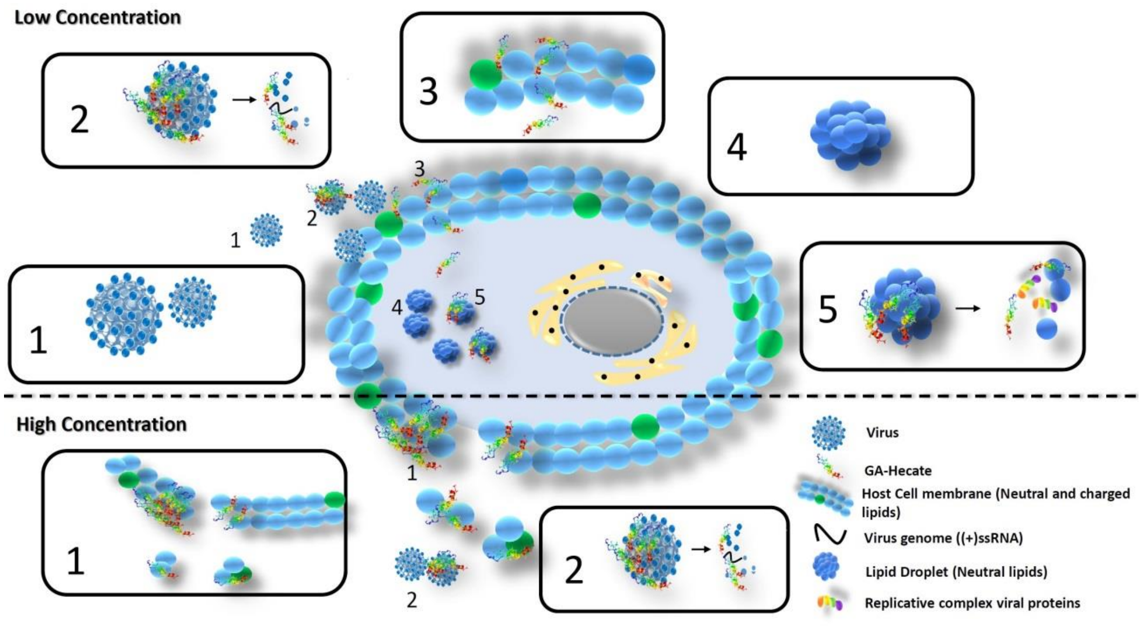

Figure S4. **GA-Hecate mechanism of action.** The proposed mechanism of action shows that at low concentrations (top panel) lipoviralparticles circulating in the medium (1) can eventually find GA-Hecate. GA-Hecate direct interaction with lipids in a small area (2) disrupts the lipid envelope and induces a virucidal effect blocking the viral entry. In low concentrations, the drug also cross the cell membrane (3) changing membrane permeability and blocking virus entry during pretreatment experiments; interacts with dsRNA and disturb lipid droplet traffic and quantity (5) inhibiting viral replication. The absence of apoptosis in low concentrations, reinforce the absence of cell death effects in low concentrations. At high concentrations (bottom panel), there are many GA-Hecate molecules which aggregate, assembling a pore structure in host cell membrane and inducing cell death by necrosis (1). In this case also the interaction with viral envelope is happening, causing a virucidal effect (2). Necrosis was the only way detected for cell death during the treatment in all tested time points reinforcing that this is the main mechanism of cell death. These results are supported by permeabilization and DLS assays which showed permeabilization effect but no change in the artificial membrane size, what is also an indicative of pore formation.

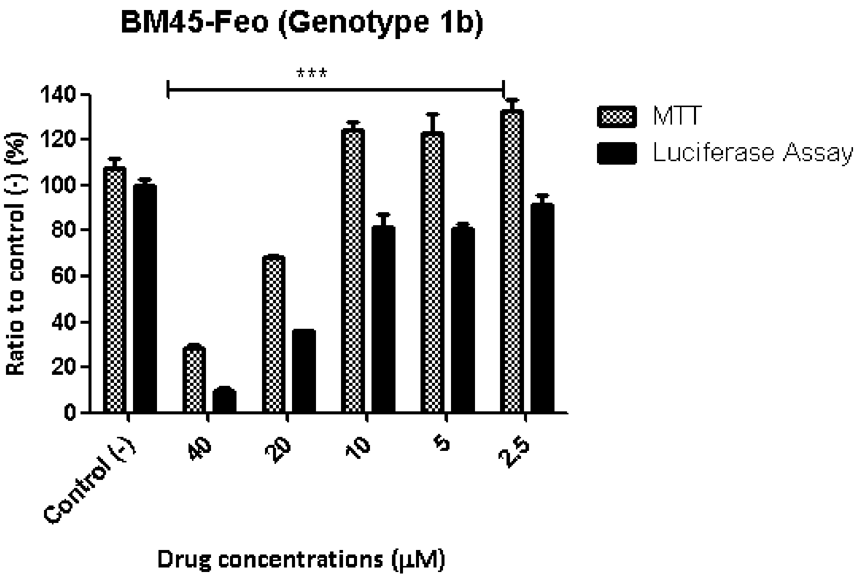

Figure S5. **GA-Hecate effect on HCV genotype 1b**. Huh-7.5 were electroporated using 2µg of BM4-5 Feo RNA and seeded in 96-well plate 24h prior to the infection. Negative control for virus inhibition was the drug solvent (nuclease free water). Bars represent the average of triplicates in two independent experiments and SD. SD: Standard deviation. *** Pvalue<0.001 vs Control (-)

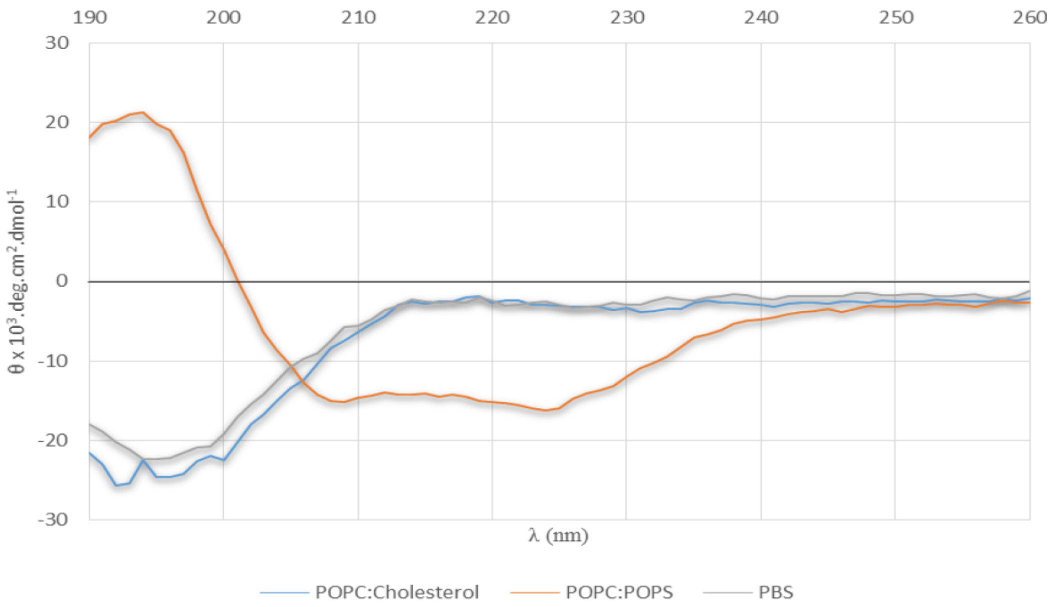

Figure S6. **Circular Dichroism of Hecate.** Circular Dichroism spectra (molar ellipticity) of Hecate in POPC:Cholesterol (neutral vesicle), POPC:POPS (negatively charged vesicles) and PBS.

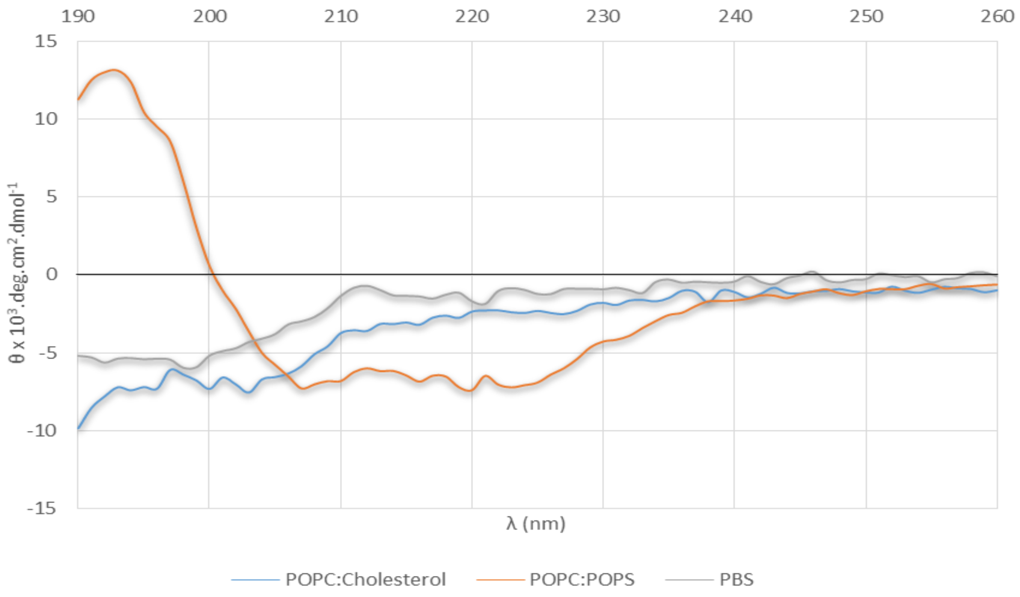

Figure S7. **Circular Dichroism of GA-Hecate.** Circular Dichroism spectra (molar ellipticity) of Hecate in POPC:Cholesterol (neutral vesicle), POPC:POPS (negatively charged vesicles) and PBS.

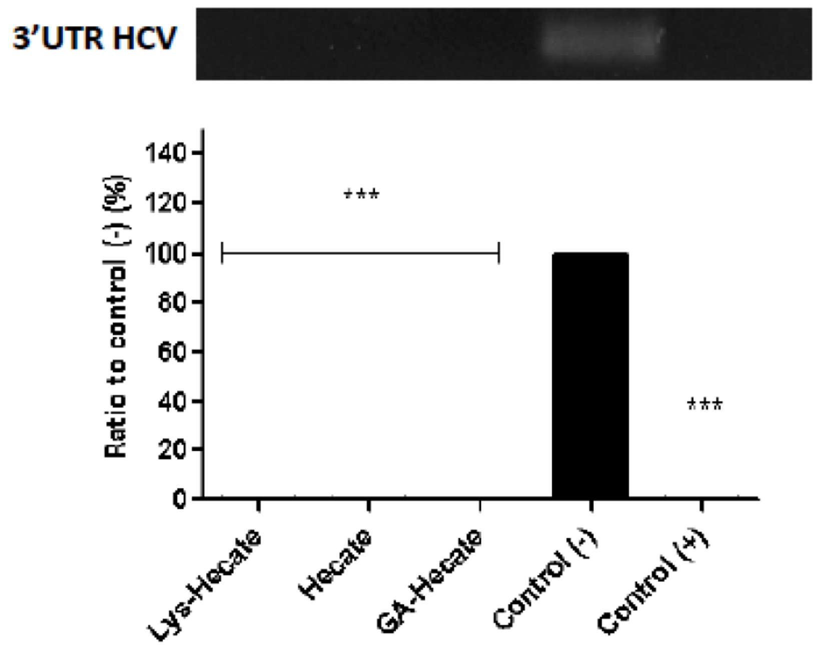

Figure S8. **dsRNA intercalation assay.** The assay shows the most positive peptide (Lys-Hecate), the least positive peptide with the steric effect (GA-Hecate) and the Hecate general structure effects on dsRNA intercalation. Water was used as a negative control for dsRNA intercalation and doxorubicin 100 µM was used as a positive control.
